# Comorbidities in congenital heart disease: different patterns in childhood and adulthood

**DOI:** 10.1186/s12872-023-03654-5

**Published:** 2023-12-13

**Authors:** Zhibao Ding, Jingai Zhu, Ye Ding, Chun Zhu

**Affiliations:** 1 Department of Pediatrics, Lishui City People’s Hospital, Zhongda Hospital Lishui Branch, Southeast University, Nanjing, 211200 Jiangsu Province People’s Republic of China; 2 Department of Pediatrics, Women’s Hospital of Nanjing Medical University, Nanjing Maternity and Child Health Care Hospital, No. 123 Tianfei Lane, Mochou Road, Nanjing, 210004 Jiangsu Province People’s Republic of China; 3 Department of Obstetrics and Gynecology, Women’s Hospital of Nanjing Medical University, Nanjing Maternity and Child Health Care Hospital, No. 123 Tianfei Lane, Mochou Road, Nanjing, 210004 Jiangsu Province People’s Republic of China; 4 Department of Child Health Care, Women’s Hospital of Nanjing Medical University, Nanjing Maternity and Child Health Care Hospital, No. 123 Tianfei Lane, Mochou Road, Nanjing, 210004 Jiangsu Province People’s Republic of China

**Keywords:** Congenital heart disease, Association rules analysis, Children, Adults, Comorbidities

## Abstract

**Background:**

Existing studies were no exploration of the association between congenital heart disease (CHD) in children and comorbidities. This study was to assess the prevalence and number of comorbidities in CHD among children and adults, and to compare the comorbidity patterns by children and adults using association rule analysis.

**Methods:**

Patients identified by the International Classification of Diseases, Ninth Revision (ICD‐9) code in the Medical Information Mart for Intensive Care III (MIMIC-III) 2001–2012 and MIMIC-IV 2008–2018 were included in this cross-sectional study. Association rule analysis was used to explore associations between CHD and comorbidities in children and adults using values of support (%), confidence (%), and lift.

**Results:**

Among 60,400 eligible patients, 1.54% of adults had CHD and 0.83% of adults had CHD with at least one comorbidity, 13.79% had CHD and 12.37% had CHD with at least one comorbidity in children. The most common comorbidities were circulatory system diseases (53.78%), endocrine diseases (35.76%), and respiratory system diseases (23.46%) in adults with CHD, and the most common comorbidities were perinatal diseases (87.50%) in children with CHD. The comorbidity rate was 90.19% and 56.68% in children and adults, respectively. In children, perinatal diseases, circulatory system diseases, and endocrine diseases had the highest prevalence. The incidence of circulatory system diseases, perinatal diseases and endocrine diseases in CHD adults was confidence = 31.56%, 36.11%, and 23.23%, respectively. Perinatal diseases were common comorbidities among all CHD severity groups in children and adults.

**Conclusion:**

The prevalence of comorbidities in children with CHD was higher than that in adults with CHD. The most common comorbidities were perinatal diseases and endocrine diseases among children and adults with CHD, respectively. Our study provided insights into comorbidity patterns in children and adults with CHD.

**Supplementary Information:**

The online version contains supplementary material available at 10.1186/s12872-023-03654-5.

## Introduction

Congenital heart disease (CHD) is the most common congenital malformation, and CHD constitutes the largest group of infants with congenital anomalies requiring neonatal surgery, affecting nearly one percent of live births each year [1, 2]. A meta-analysis of the global birth prevalence of CHD showed a gradual increase in the global prevalence of births with CHD between 1970–2017, reaching a peak value of 9.410 cases per 1,000 births in 2010–2017 [3]. With the development of clinical diagnosis and treatment techniques, the improvement of pediatric surgical and perioperative outcomes, the mortality rate of CHD patients has decreased significantly, and the number of adults with CHD has gradually increased [4, 5]. However, CHD survivors are at increased risk of developing comorbidities as they age, resulting in additional health care expenditures and loss of life, creating a significant disease burden [6]. As life expectancy increases, so does the need to understand the burden of acquired disease in the CHD patient population [7].

At present, a few studies examined the comorbidity burden of CHD in different populations. A previous study demonstrated an association between CHD and cancer [8]. Sarah et al. [9] showed that coronary heart disease was the most common cause of death in patients with severe CHD, and ischemic heart disease and malignant tumors were the most common causes of death in patients with non-severe CHD. Existing studies were limited to the association between adult CHD and certain acquired diseases, and there was no exploration of the association between CHD children and comorbidities, and the association between CHD and comorbidities was insufficient. The age and complexity of CHD were significantly correlated with the presence of complications [10], we speculated that there were differences in CHD characteristics between children and adults. However, based on current studies, it is unclear whether there are differences between children and adults. Therefore, it is necessary to further explore the comorbidity patterns of CHD patients in children and adults, and to consider the disease characteristics of CHD, to help identify high-risk populations, rationally allocate care, and improve the disease burden [11].

The purpose of this study was to analyze the comorbidity patterns of children and adults with CHD, and to further analyze the comorbidity patterns of CHD in different severities.

## Methods

### Study population

The study population in this cross-sectional study was from the Medical Information Mart for Intensive Care III (MIMIC-III) 2001–2012 and MIMIC-IV 2008–2018 (https://mimic.mit.edu/docs/iv/). MIMIC-III is a large, free-available database containing established health-related data associated with more than 40,000 patients admitted to the intensive care unit of the Beth Israel Deaconess Medical Center between 2001 and 2012 [12]. MIMIC-IV is a relational database containing actual hospital stays for patients admitted to a tertiary academic medical center in Boston, Massachusetts, the USA between 2008–2018. Of the 60,859 patients identified by the International Classification of Diseases, Ninth Revision (ICD‐9) were extracted from the database, and 459 patients were excluded due to the age information missing. Finally, 60,400 patients (43,866 patients from MIMIC-III and 16,534 patients from MIMIC-IV) were included.

The MIMIC database project was approved by the Institutional Review Boards of Beth Israel Deaconess Medical Center (Boston, Massachusetts) and the Massachusetts Institute of Technology (Cambridge, Massachusetts). All patient health information was de-identified, so our study was exempt from approval by the ethical review board of local hospital.

### Severity of CHD

According to the severity of the lesions, CHD was divided into severe CHD, shunt CHD, valve CHD and others (severe > shunt > valve > other) [9, 13]. Patients with more than one lesion were classified by the lesion of highest severity (severe CHD). Severe CHD included common truncus (ICD code 745.0), transposition of the great arteries (TGA) (ICD code 745.1), complete TGA (dextro-TGA) (ICD code 745.10), double outlet right ventricle (DORV) or incomplete TGA (ICD code 745.11), corrected TGA (levo-TGA) (ICD code 745.12), other TGA (ICD code 745.19), tetralogy of fallot (ICD code 745.2), single ventricle, or cortriloculare (ICD code 745.3), endocardial cushion defect (AVSD) (ICD code 745.6), endocardial cushion defect (AVSD) unspecified (ICD code 745.60), endocardial cushion defect, other (ICD code 745.69), pulmonary valve atresia or absence (ICD code 746.01), tricuspid atresia, stenosis or absence (ICD code 746.1), hypoplastic left heart syndrome (ICD code 746.7), congenital heart block (ICD code 746.86), interrupted aortic arch (ICD code 747.11), and total anomalous pulmonary venous return (TAPVR) (ICD code 747.41).

Shunt CHD included ventricular septal defect (ICD code 745.4),atrial septal defect (ASD) or patent foramen ovale (ICD code 745.5), ASD (primum) (ICD code 745.61), other specified defects of septal closure (ICD code 745.8), unspecified defect of septal closure (ICD code 745.9), patent ductus arteriosus (ICD code 747.0), pulmonary arteriovenous malformation (ICD code 747.32), and partial anomalous venous return (PAPVR) (ICD code 747.42).

Valve CHD included anomalies of the pulmonary valve (ICD code 746.0), pulmonary valve anomaly, unspecified (ICD code 746.00), pulmonary valve stenosis (ICD code 746.02), pulmonary valve anomaly, other (ICD code 746.09), ebstein’s anomaly (ICD code 746.2), aortic valve stenosis (ICD code 746.3), aortic insufficiency or bicuspid/unicuspid aortic valve (ICD code 746.4), mitral stenosis or mitral valve abnormalities (ICD code 746.5), mitral insufficiency (ICD code 746.6), subaortic stenosis (ICD code 746.81), infundibular or subvalvar pulmonary stenosis (ICD code 746.83), coarctation of aorta (ICD code 747.10 or 747.1), atresia or stenosis of aorta (ICD code 747.22), anomalies of pulmonary artery (ICD code 747.3), pulmonary artery atresia, coarctation, or hypoplasia (ICD code 747.31), and anomalies of pulmonary artery, other (ICD code 747.39).

Other CHD included (case only has one or more codes in this category) corbiloculare (ICD code 745.7), other specified anomalies of heart (ICD code 746.8), cortriatriatum (ICD code 746.82), obstructive anomalies of heart (ICD code 746.84), coronary artery anomaly (ICD code 746.85), malposition of heart or apex (ICD code 746.87), other specified anomaly of heart (various types) (ICD code 746.89), unspecified defect of heart (ICD code 746.9), other anomaly of the aorta (ICD code 747.2), anomalies of aorta, unspecified (ICD code 747.20), anomaly of aortic arch (ICD code 747.21), other anomaly of aorta (ICD code 747.29), anomalies of great veins (ICD code 747.4), anomalies of great veins, unspecified (ICD code 747.40), other anomalies of great veins (ICD code 747.49), unspecified anomalies of circulatory system (ICD code 747.9), congenital cardiovascular disorder complicating pregnancy, childbirth or puerperium (ICD code 648.5X), and personal history of corrected congenital malformations of the heart and circulatory system (ICD code V13.65).

### Data collection

All data were obtained from the medical records of hospitalized patients. For patients who were admitted to the ICU more than once, only data from the patient’s first ICU admission were used for analysis. Demographic variables of age, gender (male or female) and ethnicity (Asian, Black, White, or other) were collected. According to ICD-9, the comorbidities we collected included infectious diseases (ICD codes 001–1396), neoplasms (ICD codes 140–239), blood diseases (ICD codes 2800–2899), endocrine diseases (ICD codes 2400–2788), mental disorders (ICD codes 2900–319), nervous system diseases (ICD codes 320–359), eye and adnexa diseases (ICD codes 360–379), ear and mastoid process diseases (ICD codes 380–389), circulatory system diseases (ICD codes 390–459), respiratory system diseases (ICD codes 460–519), digestive system diseases (ICD codes 520–579), skin and subcutaneous tissue diseases (ICD codes 680–709), musculoskeletal system diseases (ICD codes 710–739), genitourinary system diseases (ICD codes 580–629), pregnancy, childbirth, and puerperium complications (ICD codes 630–679), perinatal diseases (ICD codes 760–779), and other congenital anomalies [ICD codes 740–759(9) exclude CHD].

### Statistical analysis

Median and quartile [M (Q1, Q3)] was used to describe the distribution of continuous variable that did not follow the normal distribution, and the Wilcoxon rank sum test was used to compare the difference between groups. The number of frequencies and percentages [n (%)] were used to describe the distribution of categorical variable, and the chi-square test was used to compare the differences between groups. First, the study population was divided into two groups, adults and children, for descriptive statistical analysis. Second, we divided CHD patients into two groups children and adults, to explore the characteristics of different groups and analyze the prevalence of CHD comorbidities and the number of CHD comorbidities in children and adults. Third, Association rule analysis was used to explore associations between CHD and comorbidities in children and adults using values of support (%), confidence (%), and lift [14]. Association rule analysis is a machine learning algorithm that automatically discovers potential rules or patterns from data to describe associations, dependencies, or other useful information in the data. This type of analysis relies on a measure of “interestingness” a term related to the effect size of a pattern, rather than a simple test of statistical significance [14, 15]. The results in this study are presented in terms of support (%), confidence (%), and lift values, which reflect the probability and association of different comorbidities with the occurrence of CHD. The “support” value is the probability of simultaneous occurrence of CHD and certain comorbidity. The “confidence” value indicates the ratio of occurrence of disease a comorbidity and CHD at the same time a comorbidity occurs [16]. The “lift” value reflects the correlation between CHD and comorbidities in the association rules. Lift > 1 indicates a high positive correlation, lift < 1 indicates a high negative correlation, and lift = 1 indicates no correlation between CHD and comorbidities. At last, associations between different severity of CHD and comorbidities in children and adults were explored.

SAS v. 9.4 (SAS Institute, Cary, North Carolina) was used for descriptive statistics and comparison between groups. Association rule analysis was performed by R v. 4.0.3 (R Foundation for Statistical Computing, Vienna, Austria), R arules and R aruleViz were used for analysis and visualization of network analysis, and Python v. 3.8.3 (Python Software Foundation, DE, USA) was used for correlation analysis.

### Data availability statement

The data used to support the findings of this study are mainly included within the article, and the underlying data are available from the corresponding author upon request.

## Results

### Characteristics of the study population

Among 60,400 eligible patients, the median age of children was 0.00 years, and the median age of adults was 65.84 years (Supplementary Table 1). In the adults, 843 (1.54%) had CHD, 390 (0.71%) had CHD alone, and 453 (0.83%) had CHD with at least one comorbidity. Among the children, 790 (13.79%) had CHD, 81 (1.41%) had CHD alone, and 709 (12.37%) had CHD with at least one comorbidity.

Table 1 presents the characteristics of CHD patients in the total study population. There were significant differences in age (*P* < 0.001), gender (*P* < 0.001), ethnicity (*P* < 0.001), and severity of CHD (*P* < 0.001) between children with CHD and adults with CHD. The number of male patients was higher than that of females in children with CHD (52.28% vs. 47.72%, *P* < 0.001) and adults with CHD (69.63% vs. 30.37%, *P* < 0.001). In terms of CHD severity, shunt CHD was most common in children [530 (67.09%)], whereas valve CHD was most common in adults [607 (72.00%)].

**Table 1:** Characteristics of children and adults with congenital heart disease (CHD)

Variables	Children (*n* = 790)	Adults (*n* = 843)	Statistics	*P*
Age, years, M (Q_1_, Q_3_)	0.00 (0.00,0.00)	57.60 (49.00, 66.05)	Z = -36.508	< 0.001
Gender, n (%)			χ^2^ = 51.740	< 0.001
Female	377 (47.72)	256 (30.37)		
Male	413 (52.28)	587 (69.63)		
Ethnicity, n (%)			χ^2^ = 82.551	< 0.001
Asian	57 (7.22)	13 (1.54)		
Black	92 (11.64)	31 (3.68)		
White	483 (61.14)	654 (77.58)		
Other	158 (20.00)	145 (17.20)		
Severity of CHD, n (%)			χ^2^ = 624.040	< 0.001
Severe CHD	59 (7.47)	22 (2.61)		
Shunt CHD	530 (67.09)	92 (10.91)		
Valve CHD	167 (21.14)	607 (72.00)		
Other CHD	34 (4.30)	122 (14.47)		

The children and adults were divided into three groups non-CHD, CHD alone and CHD with at least one comorbidity. The results of the comparison between the groups are shown in Table 2. Significant differences were found in age (*P* < 0.001) in children, and in age (*P* < 0.001), gender (*P* < 0.001) and ethnicity (*P* < 0.001) in adults.

**Table 2:** Results of the comparison between the groups non-CHD, CHD alone and CHD with at least one comorbidity in children and adults

Variables	Non-CHD (*n* = 4940)	CHD alone (*n* = 81)	CHD with at least one comorbidity (*n* = 709)	Statistics	*P*
**Children**					
Age, years, M (Q_1_, Q_3_)	0.00 (0.00,0.00)	0.00 (0.00,0.01)	0.00 (0.00,0.00)	χ^2^ = 55.148	< 0.001
Gender, n (%)				χ^2^ = 3.588	0.166
Female	2,183 (44.19)	37 (45.68)	340 (47.95)		
Male	2,757 (55.81)	44 (54.32)	369 (52.05)		
Ethnicity, n (%)				χ^2^ = 8.682	0.192
Asian	383 (7.75)	8 (9.88)	49 (6.91)		
Black	533 (10.79)	7 (8.64)	85 (11.99)		
White	3,115 (63.06)	57 (70.37)	426 (60.08)		
Other	909 (18.40)	9 (11.11)	149 (21.02)		
**Adults**					
Age, years, M (Q_1_, Q_3_)	66.00 (53.00,77.84)	59.00 (50.00,67.00)	57.04 (48.07,65.59)	χ^2^ = 198.387	< 0.001
Gender, n (%)				χ^2^ = 52.315	< 0.001
Female	23,013 (42.75)	115 (29.49)	141 (31.13)		
Male	30,814 (57.25)	275 (70.51)	312 (68.87)		
Ethnicity, n (%)				χ^2^ = 26.941	< 0.001
Asian	1,296 (2.41)	7 (1.79)	6 (1.32)		
Black	4,144 (7.70)	16 (4.10)	15 (3.31)		
White	38,210 (70.99)	301 (77.18)	353 (77.92)		
Other	10,177 (18.91)	66 (16.92)	79 (17.44)		

*CHD* Congenital heart disease

### Prevalence and number of comorbidities in children and adults with CHD

**Fig. 1:**
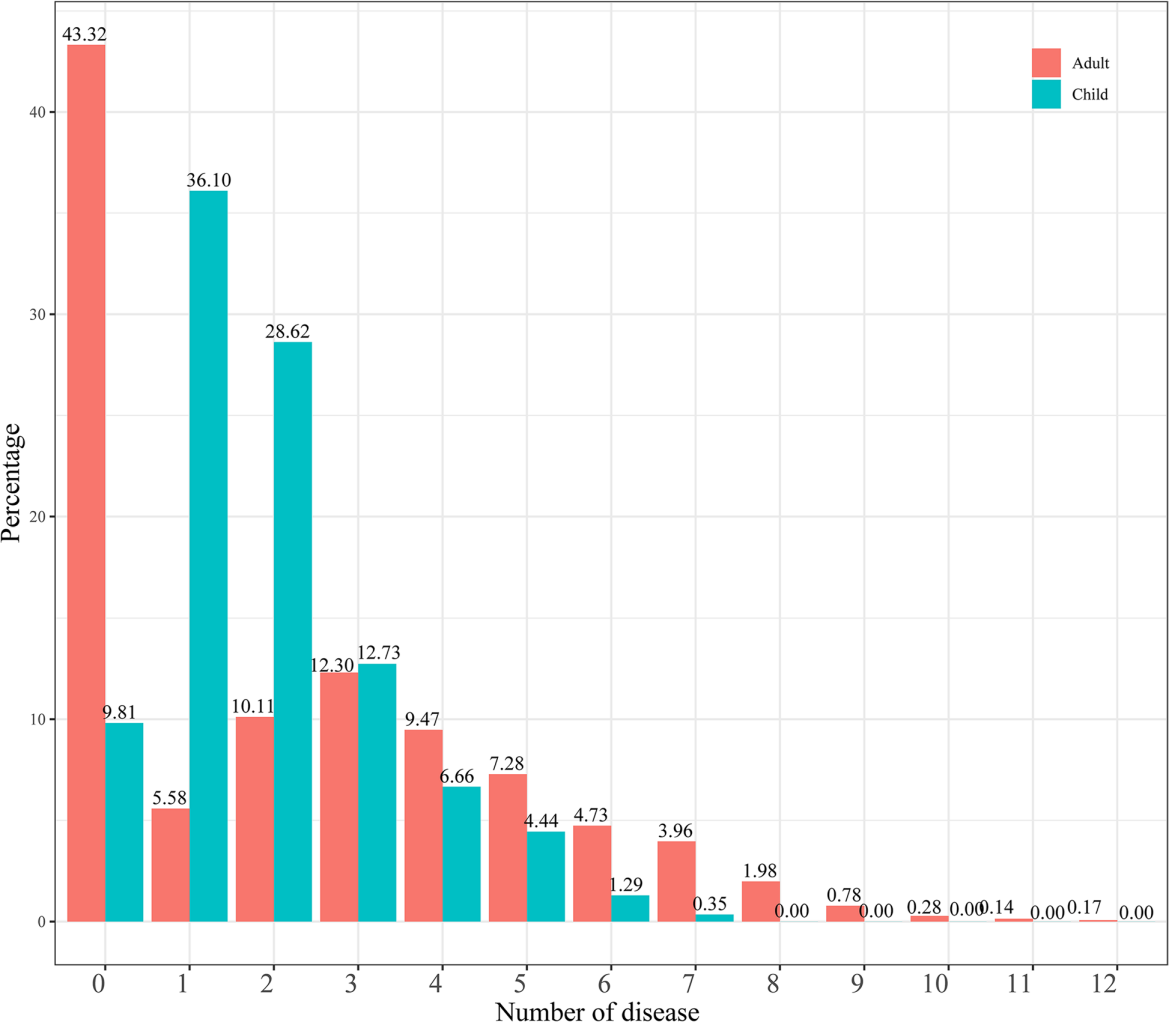
Number of comorbidities in children and adults with CHD

The most common comorbidities were circulatory system diseases [53.78% (95CI: 51.18–56.36)], endocrine diseases [35.76% (95CI: 33.30–38.29)], and respiratory system diseases [23.46% (95CI: 21.33–25.74)] in adults with CHD, and the most common comorbidities were perinatal diseases [87.50% (95CI: 85.16–89.59)], pregnancy, childbirth, and puerperium complications [23.36% (95CI: 20.65–26.31)], and other congenital anomalies [22.08% (95CI: 19.43–24.98)] in children with CHD (Table 3). As Fig. 1 shows, 9.81% of CHD children had no comorbidities, and the comorbidity rate was 90.19%. One comorbidity was present in 36.10% of the CHD children, two comorbidities were present in 28.62% of the CHD children, the largest number of comorbidities was seven. There were 43.32% of CHD adults had no comorbidities, the maximum comorbidity was twelve in CHD adults.

**Table 3:** Prevalence of comorbidities in children and adults with congenital heart disease (CHD)

Comorbidities	Children	Adults
	**Prevalence % (95% CI)**	**Prevalence % (95% CI)**
Perinatal diseases	87.50 (85.16–89.59)	0.00 (0.00–0.00)
Pregnancy, childbirth, and puerperium complications	23.36 (20.65–26.31)	0.14 (0.04–0.51)
Other congenital anomalies	22.08 (19.43–24.98)	1.77 (1.20–2.60)
Eye and adnexa diseases	12.50 (10.45–14.88)	3.18 (2.39–4.23)
Digestive system diseases	12.50 (10.45–14.88)	17.88 (15.97–19.96)
Circulatory system diseases	9.00 (7.26–11.10)	53.78 (51.18–56.36)
Skin and subcutaneous tissue diseases	5.37 (4.05–7.09)	5.09 (4.06–6.36)
Endocrine diseases	4.67 (3.45–6.30)	35.76 (33.30–38.29)
Respiratory system diseases	3.74 (2.66–5.23)	23.46 (21.33–25.74)
Nervous system diseases	3.15 (2.18–4.55)	13.36 (11.68–15.23)
Genitourinary system diseases	1.99 (1.24–3.16)	16.61 (14.76–18.64)
Neoplasms	1.52 (0.89–2.58)	4.31 (3.37–5.50)
Blood disease	1.40 (0.80–2.43)	18.73 (16.78–20.84)
Musculoskeletal system diseases	1.17 (0.64–2.14)	9.82 (8.38–11.48)
Infectious diseases	0.47 (0.18–1.20)	1.27 (0.81–2.00)
Mental disorders	0.12 (0.02–0.66)	15.12 (13.35–17.08)
Ear and mastoid process diseases	0.00 (0.00–0.00)	0.85 (0.49–1.48)

### Association between CHD and comorbidities in children and adults

**Fig. 2:**
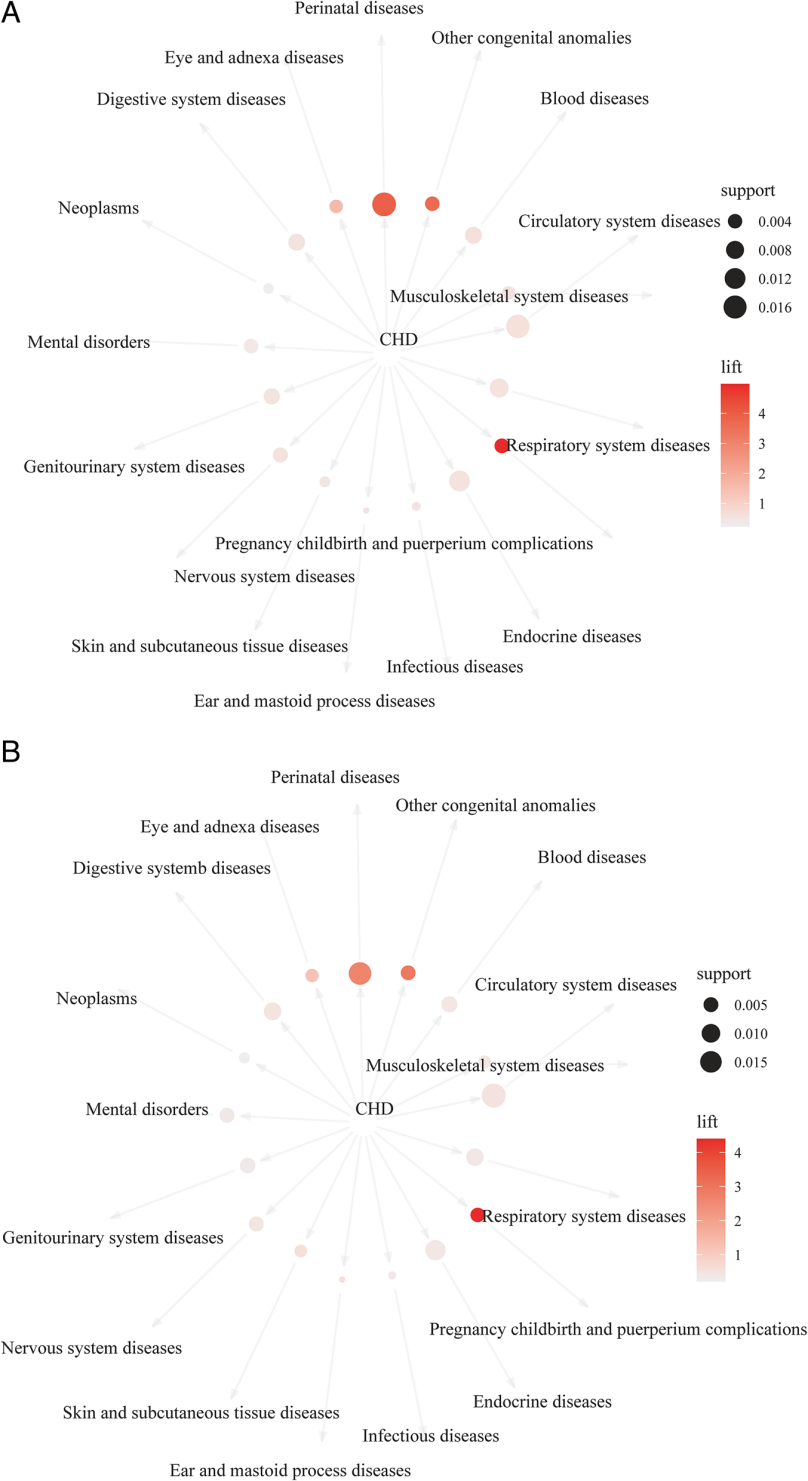
Network diagram of CHD and comorbidities in children (**A**) and adults (**B**)

Table 4 shows the association rules analysis of CHD and comorbidities in children and adults. In children, compared with other comorbidities, children with CHD had the highest probability of comorbidity with perinatal diseases, circulatory system diseases, and endocrine diseases, and the value of support was 1.72%, 1.63%, and 1.17%, respectively. 47.55% of children with perinatal diseases, 45.10% of children with circulatory system diseases, and 32.35% of children with endocrine diseases also reported CHD, which was reflected by the confidence value. Under the condition of CHD, the probability of perinatal diseases increased by 3.94 times (lift = 3.94), and the probabilities of circulatory system diseases (lift = 0.62) and endocrine diseases (lift = 0.55) were decreased (lift < 1). It could also be seen from Table 4 that among CHD children, the probability of suffering from pregnancy, childbirth and puerperium complications, other congenital anomalies, and eye and adnexa diseases increased by 4.98, 3.65, and 1.58 times, respectively. Network diagram Fig. 2A is a visual display of the association between CHD and comorbidities in children, and the results were consistent with Table 4.

**Table 4:** Association rules analysis of CHD and comorbidities in children and adults

LHS	RHS	Children			Adults		
		**Support (%)**	**Confidence (%)**	**Lift**	**Support (%)**	**Confidence (%)**	**Lift**
CHD	Perinatal diseases	1.72	47.55	3.94	1.71	31.56	2.64
CHD	Circulatory system diseases	1.63	45.10	0.62	1.95	36.11	0.50
CHD	Endocrine diseases	1.17	32.35	0.55	1.26	23.23	0.40
CHD	Respiratory system diseases	0.91	25.00	0.58	0.82	15.15	0.36
CHD	Blood diseases	0.64	17.65	0.59	0.63	11.67	0.38
CHD	Digestive system diseases	0.64	17.65	0.53	0.85	15.68	0.46
CHD	Genitourinary system diseases	0.59	16.18	0.48	0.57	10.60	0.31
CHD	Nervous system diseases	0.44	12.25	0.55	0.50	9.24	0.42
CHD	Pregnancy, childbirth, and puerperium complications	0.43	11.76	4.98	0.47	8.62	4.40
CHD	Other congenital anomalies	0.41	11.27	3.65	0.50	9.24	2.90
CHD	Mental disorders	0.41	11.27	0.42	0.50	9.29	0.33
CHD	Eye and adnexa diseases	0.32	8.82	1.58	0.35	6.49	1.21
CHD	Musculoskeletal system diseases	0.32	8.82	0.52	0.34	6.34	0.40
CHD	Skin and subcutaneous tissue diseases	0.14	3.92	0.45	0.29	5.32	0.59
CHD	Neoplasms	0.12	3.43	0.23	0.18	3.24	0.22
CHD	Infectious diseases	0.05	1.47	0.52	0.05	0.92	0.33
CHD	Ear and mastoid process diseases	0.02	0.49	0.51	0.03	0.53	0.58

*CHD* Congenital heart disease, *LHS* Left-hand-side, *RHS* Right-hand-side

In the adult population, CHD comorbidities with perinatal diseases (support = 1.71%), circulatory system diseases (1.95%), and endocrine diseases (1.26%) were the highest, compared with CHD and other comorbidities (Table 4 and Fig. 2B). The incidence of circulatory system diseases, perinatal diseases and endocrine diseases in CHD adults was confidence = 31.56%, 36.11%, and 23.23%, respectively, but, circulatory system diseases and endocrine diseases rates declined (lift < 1), while perinatal rates increased 2.64 times (lift = 2.64). Under the condition of CHD, the probability of pregnancy, childbirth and puerperium complications (lift = 4.40), other congenital anomalies (lift = 2.90), and eye and adnexa diseases (lift = 1.21) was increased. Figure 2B is the network diagram of CHD and comorbidities in adults.

### Association between severe, shunt and valve CHD and comorbidities in children

**Fig. 3:**
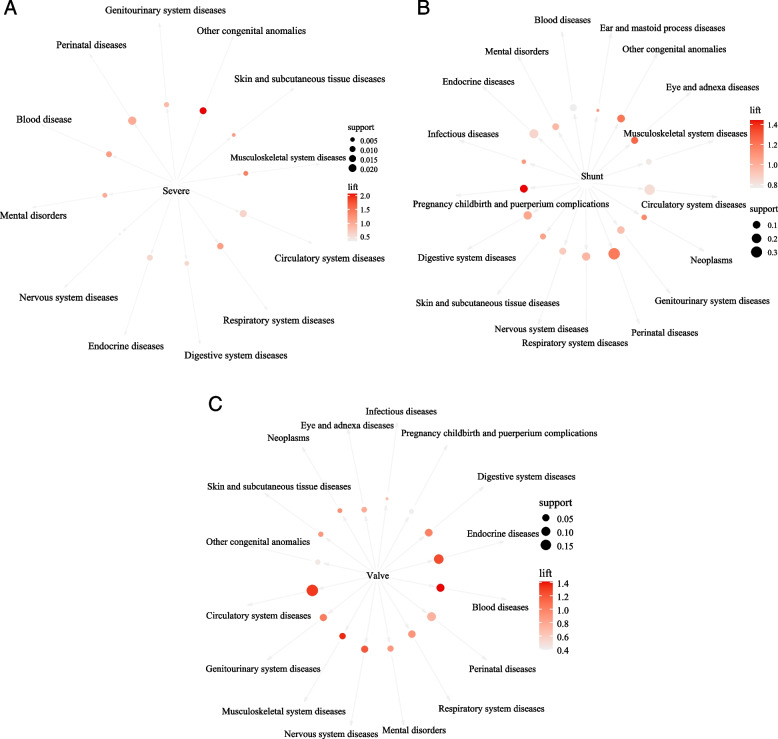
Network diagram of severe CHD and comorbidities (**A**), shunt CHD and comorbidities (**B**), and valve CHD and comorbidities (**C**) in children

In Table 5 and Fig. 3A, the probability of suffering from perinatal diseases was the highest (confidence = 45.24%), and the probabilities of suffering from other congenital anomalies (lift = 2.07), respiratory system diseases (lift = 1.12), blood diseases (lift = 1.14), musculoskeletal system diseases (lift = 1.40), skin and subcutaneous tissue diseases (lift = 1.13) were increased among children with severe CHD.

**Table 5:** Association rules analysis of different severity CHD and comorbidities in children

LHS	RHS	Support (%)	Confidence (%)	Lift
Severe CHD	Perinatal diseases	2.22	45.24	1.01
	Circulatory system diseases	1.52	30.95	0.62
	Other congenital anomalies	1.40	28.57	2.07
	Respiratory system diseases	1.17	23.81	1.12
	Blood diseases	0.93	19.05	1.14
	Endocrine diseases	0.93	19.05	0.59
	Genitourinary system diseases	0.70	14.29	0.87
	Musculoskeletal system diseases	0.58	11.90	1.40
	Mental disorders	0.58	11.90	1.01
	Digestive system diseases	0.58	11.90	0.57
	Skin and subcutaneous tissue diseases	0.35	7.14	1.13
	Nervous system diseases	0.23	4.76	0.36
Shunt CHD	Perinatal diseases	32.83	53.52	1.19
	Circulatory system diseases	25.47	41.52	0.83
	Endocrine diseases	17.06	27.81	0.86
	Digestive system diseases	13.20	21.52	1.04
	Respiratory system diseases	12.73	20.76	0.98
	Pregnancy, childbirth, and puerperium complications	10.63	17.33	1.44
	Other congenital anomalies	10.16	16.57	1.20
	Genitourinary system diseases	9.46	15.43	0.94
	Blood diseases	7.83	12.76	0.76
	Eye and adnexa diseases	7.48	12.19	1.26
	Nervous system diseases	7.36	12.00	0.90
	Mental disorders	7.01	11.43	0.97
	Skin and subcutaneous tissue disease	4.09	6.67	1.06
	Musculoskeletal system disease	4.09	6.67	0.78
	Neoplasms	3.04	4.95	1.15
	Infectious diseases	1.17	1.90	1.09
	Ear and mastoid process diseases	0.23	0.38	1.09
Valve CHD	Circulatory system diseases	19.16	65.60	1.32
	Endocrine diseases	11.80	40.40	1.25
	Perinatal diseases	9.58	32.80	0.73
	Blood diseases	6.89	23.60	1.41
	Digestive system diseases	6.07	20.80	1.00
	Respiratory system diseases	5.61	19.20	0.90
	Genitourinary system diseases	4.91	16.80	1.03
	Nervous system diseases	4.67	16.00	1.20
	Musculoskeletal system diseases	3.39	11.60	1.36
	Mental disorders	3.04	10.40	0.88
	Eye and adnexa diseases	2.22	7.60	0.78
	Other congenital anomalies	1.75	6.00	0.44
	Skin and subcutaneous tissue diseases	1.64	5.60	0.89
	Pregnancy, childbirth and puerperium complications	1.40	4.80	0.40
	Neoplasms	1.17	4.00	0.93
	Infectious diseases	0.35	1.20	0.68

*CHD* Congenital heart disease, *LHS* Left-hand-side, *RHS* Right-hand-side

The comorbidities of shunt CHD, perinatal diseases and circulatory system diseases accounted for 32.83% and 25.47%. Under the condition of shunt CHD, the probability of suffering from perinatal diseases and circulatory system diseases was 53.52% and 41.52%, respectively. The probabilities of perinatal diseases (lift = 1.19), pregnancy, childbirth, and puerperium complications (lift = 1.44), other congenital anomalies (lift = 1.20), eye and adnexa diseases (lift = 1.26), neoplasms (lift = 1.15) were increased in children with shunt CHD (Table 5 and Fig. 3B).

The probabilities of circulatory system diseases and endocrine diseases in children with valve CHD were 65.60% and 40.40%. In children with valve CHD, the probability of suffering from circulatory system diseases, endocrine diseases, blood diseases, nervous system diseases, and musculoskeletal system diseases has increased (lift > 1) (Table 5 and Fig. 3C).

### Association between severe, shunt and valve CHD and comorbidities in adults

**Fig. 4:**
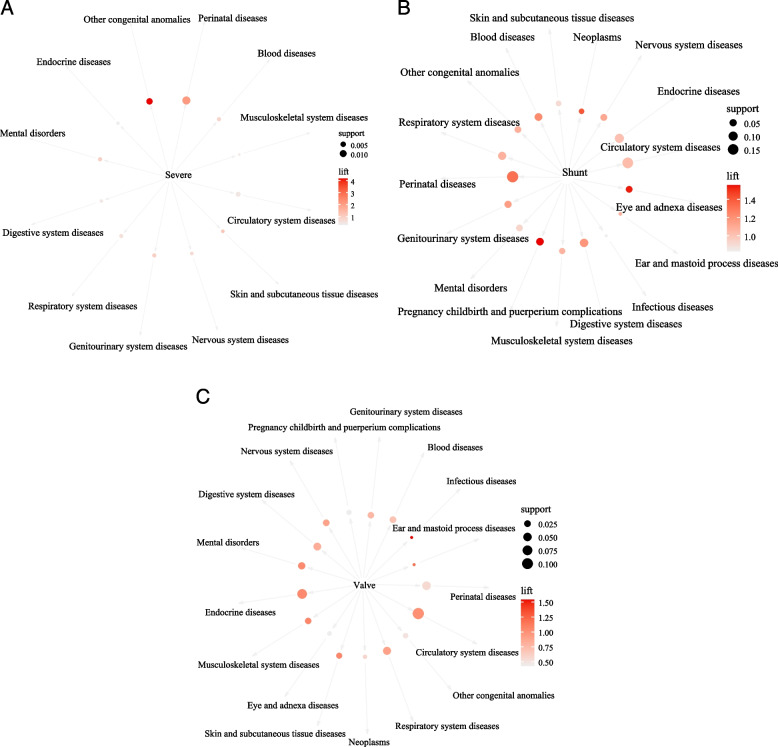
Network diagram of severe CHD and comorbidities (**A**), shunt CHD and comorbidities (**B**), and valve CHD and comorbidities (**C**) in adults

In adults with severe CHD, the probability of perinatal diseases was 53.85%. There were positive correlations between CHD and perinatal diseases (lift = 2.09), other congenital anomalies (lift = 4.16), and skin and subcutaneous tissue diseases (lift = 1.13) (Table 6 and Fig. 4A).

**Table 6:** Association rules analysis of different severity CHD and comorbidities in adults

LHS	RHS	Support (%)	Confidence (%)	Lift
Severe CHD	Perinatal diseases	1.48	53.85	2.09
	Other congenital anomalies	0.78	28.21	4.16
	Circulatory system diseases	0.35	12.82	0.44
	Genitourinary system diseases	0.21	7.69	0.97
	Mental disorders	0.21	7.69	0.95
	Blood diseases	0.21	7.69	0.81
	Respiratory system diseases	0.21	7.69	0.60
	Skin and subcutaneous tissue diseases	0.14	5.13	1.13
	Nervous system diseases	0.14	5.13	0.71
	Digestive system diseases	0.14	5.13	0.40
	Endocrine diseases	0.14	5.13	0.27
	Musculoskeletal system diseases	0.07	2.56	0.48
Shunt CHD	Perinatal diseases	17.60	33.88	1.32
	Circulatory system diseases	15.55	29.93	1.03
	Endocrine diseases	10.04	19.32	1.02
	Digestive system diseases	7.92	15.24	1.18
	Respiratory system diseases	7.14	13.74	1.07
	Blood diseases	6.01	11.56	1.22
	Pregnancy, childbirth, and puerperium complications	5.65	10.88	1.56
	Genitourinary system diseases	4.73	9.12	1.15
	Nervous system diseases	4.17	8.03	1.11
	Eye and adnexa diseases	3.89	7.48	1.53
	Mental disorders	3.89	7.48	0.93
	Other congenital anomalies	3.82	7.35	1.08
	Musculoskeletal system diseases	2.97	5.71	1.06
	Skin and subcutaneous tissue diseases	2.12	4.08	0.90
	Neoplasms	1.91	3.67	1.40
	Ear and mastoid process diseases	0.35	0.68	1.07
	Infectious diseases	0.21	0.41	0.83
Valve CHD	Circulatory system diseases	10.81	29.20	1.01
	Endocrine diseases	7.35	19.85	1.04
	Perinatal diseases	5.30	14.31	0.56
	Respiratory system diseases	4.31	11.64	0.91
	Digestive system diseases	3.96	10.69	0.83
	Mental disorders	3.11	8.40	1.04
	Blood diseases	2.47	6.68	0.71
	Nervous system diseases	2.40	6.49	0.90
	Genitourinary system diseases	2.26	6.11	0.77
	Musculoskeletal system diseases	2.12	5.73	1.07
	Skin and subcutaneous tissue diseases	1.77	4.77	1.05
	Other congenital anomalies	1.27	3.44	0.51
	Pregnancy, childbirth, and puerperium complications	1.13	3.05	0.44
	Eye and adnexa diseases	0.78	2.10	0.43
	Neoplasms	0.57	1.53	0.58
	Infectious diseases	0.28	0.76	1.54
	Ear and mastoid process diseases	0.28	0.76	1.20

*CHD* Congenital heart disease, *LHS* Left-hand-side, *RHS* Right-hand-side

The probabilities of perinatal diseases and circulatory system diseases were 33.88% and 29.93% in adults with shunt CHD, respectively. Positive correlations between CHD and perinatal diseases (lift = 1.32), digestive system diseases (lift = 1.18), blood diseases (lift = 1.22), pregnancy, childbirth, and puerperium complications (lift = 1.56), genitourinary system diseases (lift = 1.15), nervous system diseases (lift = 1.11), eye and adnexa diseases (lift = 1.53), and neoplasms (lift = 1.40) were found in Table 6 and Fig. 4B.

Positive correlations between CHD and infectious diseases, and between CHD and ear and mastoid process diseases were found among children with valve CHD (Table 6 and Fig. 4C).

## Discussion

In this study, results showed that comorbidities in CHD were more common in children than in adults. The highest prevalence of comorbidities was perinatal diseases (87.50%) in children, and circulatory system diseases (53.78%) in adults. Among children, the most common comorbidities with CHD were perinatal diseases, and circulatory system diseases in adults. In the subgroup of severe and shunt CHD children, perinatal diseases and circulatory system diseases were the top two diseases of degree centrality. In the subgroup of severe and shunt CHD adult, perinatal diseases were common comorbid with CHD. When the severity is valve CHD among children and adults, circulatory system diseases and endocrine diseases were common comorbid with CHD.

From the perspective of comorbidity prevalence, the comorbidity rate of CHD children was about 90.19%, and most of the children (64.72%) had 1–2 comorbidities, and the maximum number of comorbidities was 7. About half of adult CHD patients did not have comorbidities (43.32%). Among adults with CHD with comorbidities, more patients had three comorbidities, accounting for 12.30%. The maximum number of comorbidities was 12, which was greater than the maximum number of comorbidities in children. These results suggested that clinicals should treat the comorbidity intervention of CHD children and adults differently. The comorbidity rate of CHD children was higher than that of adults with CHD. Clinicians should pay attention to timely monitoring and prevention of possible high prevalence of comorbidities. In adults with CHD, special attention should be paid to interventions for many different comorbidities.

We found that the most common comorbidities were perinatal diseases (87.50%) in children with CHD, and 47.55% of children with perinatal diseases also reported CHD. A study showed that newborns with CHD were more likely to be born prematurely and/or small for gestational age than those without CHD [17], which was consistent with our findings. According to clinical experience, the symptoms of CHD patients are shortness of breath, heart murmur, low body weight, and repeated respiratory tract infections [18]. Similar results were found in our study, 45.10% of children with circulatory system diseases also reported CHD. Children with CHD will have different effects depending on the severity of their condition [19]. The mild CHD has little or no impact, the severe CHD has a serious impact on the patients. It is easy to have pneumonia complicated by acidosis, heart failure, etc., or even die when there is no timely intervention and treatment [20]. In particular, some children with single ventricle disease, such as children with hypoplastic left heart syndrome, are often accompanied by neurological developmental disorders, and early detection and treatment can help improve the prognosis of the neurological system [21]. In our study, although no association was found between children with CHD of varying severity and neurological diseases, we found that the probability of suffering from perinatal diseases was the highest in severe and shunt CHD and the probability of circulatory system diseases was the highest in children with valve CHD. The goal of neonatal CHD screening is to identify its onset status early, conduct scientific analysis and determine appropriate treatment options. Tracking and monitoring of children with CHD can better reflect the progress of CHD or other possible complications in children [6], but it should be noted that the possible comorbidities that need to be focused on in children with CHD of different severity are slightly different. A collaborative approach to care between cardiac intensive care, cardiology, and neonatology may lead to the best outcomes for children with CHD [22].

We found that the most common comorbidities were circulatory system diseases (53.78%), endocrine diseases (35.76%), and respiratory system diseases (23.46%) in adults with CHD, the results were consistent with other studies. The study by Maurer et al. ^9^ stated that the most common acquired comorbidities in adults with CHD were endocrine and metabolic diseases (30.4%), and circulatory system diseases (28.2%). Cardiovascular risk factors such as diabetes and hyperlipidemia contribute to the development of these comorbidities [23]. Renal insufficiency is an important predictor of prognosis in patients with CHD, and patients with moderate or severe renal insufficiency have a threefold higher mortality rate than normal [24]. Compared with the general population, adults with CHD are more likely to report cardiovascular comorbidities, such as a history of congestive heart failure and stroke, especially in patients with severe CHD [13]. These results suggested that clinicians could prevent circulatory system diseases, endocrine diseases, and respiratory system diseases especially in adult CHD patients.

The strengths of this study were as follows. Firstly, MIMIC-III and MIMIC-IV databases with a large sample size were used in this study, and the clinical diagnosis of all congenital and acquired diseases was based on the unified criteria of ICD-9. Secondly, the differences in CHD characteristics between children and adults were considered for stratified analysis and comparison. In addition, the characteristics of CHD were considered and the comorbidity patterns of CHD with different severity were discussed, providing a more accurate basis for clinical diagnosis, treatment and nursing. Thirdly, the study explored the potential association of diseases from the database through association rule analysis and evaluated the degree of association from three perspectives: support, confidence and lift. Finally, the eclat algorithm was used to analyze association rules in our study, and the vertical database structure is different from the traditional mining algorithm, which improves the efficiency of mining association rules.

However, a few limitations were in our study. First, the study population in this study were all identified as ICD-9 patients from the MIMIC database, so the comorbidity of CHD may not represent the general population. Second, this study was retrospective, and there may be omissions or even misclassifications of disease information obtained from medical records. Third, the eclat algorithm may affect the efficiency of the Tidset algorithm due to the huge size and consume a large amount of memory of the system, which has high requirements on hardware devices in practical applications. Fourth, this study was unable to establish a causal relationship between CHD and comorbidities. Other longitudinal studies could be conducted to explore the specific comorbidities of CHD and the possible influencing factors, to reduce the occurrence of frequent diseases.

## Conclusion

The prevalence of comorbidities in children with CHD was higher than that in adults with CHD, and the number of comorbidities in adults with CHD was greater than that in children with CHD. For children with CHD, clinicians should pay special attention to the prevention and intervention of perinatal related diseases, and for adult patients with CHD, clinicians should focus on endocrine diseases. Our study provided insights into comorbidity patterns in children and adults with CHD.

### Supplementary Information

**Additional file 1.**

## Data Availability

The datasets generated and/or analyzed during the current study are available in the Medical Information Mart for Intensive Care III and IV (MIMIC-III and -IV) repository (https://mimic.mit.edu/docs/iv/).

## Abbreviations

CHD: Congenital heart disease
MIMIC: Medical Information Mart for Intensive Care
