# Supplementary material for: Comorbidities in congenital heart disease: different patterns in childhood and adulthood
Source: BMC Cardiovasc Disord. 2023 Dec 13;23:613. doi: 10.1186/s12872-023-03654-5 (PMC10720097; doi:10.1186/s12872-023-03654-5)
Supplement: Supplementary file 1 — Additional file 1. [file 12872_2023_3654_MOESM1_ESM.docx]

**Supplementary Table 1**. Characteristics of the study population.

| **Variables** | **Children (n=5730)** | **Adults (n=54670)** | **Statistics** | ***P*** |
| --- | --- | --- | --- | --- |
| Age, year, M (Q_1_, Q_3_) | 0.00 (0.00,0.00) | 65.84 (53.00,77.65) | Z=-124.777 | <0.001 |
| Gender, n (%) |  |  | χ^2^=9.474 | 0.002 |
| Female | 2,560 (44.68) | 23,269 (42.56) |  |  |
| Male | 3,170 (55.32) | 31,401 (57.44) |  |  |
| Ethnicity, n (%) |  |  | χ^2^=620.954 | <0.001 |
| Asian | 440 (7.68) | 1,309 (2.39) |  |  |
| Black | 625 (10.91) | 4,175 (7.64) |  |  |
| White | 3,598 (62.79) | 38,864 (71.09) |  |  |
| Other | 1,067 (18.62) | 10,322 (18.88) |  |  |
| CHD, n (%) |  |  | χ^2^=2956.283 | <0.001 |
| No | 4,940 (86.21) | 53,827 (98.46) |  |  |
| Yes | 790 (13.79) | 843 (1.54) |  |  |
| CHD classification, n (%) |  |  | χ^2^=3705.689 | <0.001 |
| Non-CHD | 4,940 (86.21) | 53,827 (98.46) |  |  |
| CHD alone | 81 (1.41) | 390 (0.71) |  |  |
| CHD with at least one comorbidity | 709 (12.37) | 453 (0.83) |  |  |

Note: CHD, congenital heart disease.
